# Effectiveness of a New Recombinant antiGnRH Vaccine for Immunocastration in Bulls

**DOI:** 10.3390/ani11051359

**Published:** 2021-05-11

**Authors:** Paula R. Huenchullan, Sonia Vidal, Rafael Larraín, Leonardo Saénz

**Affiliations:** 1 Programa de Doctorado en Ciencias Silvoagropecuarias y Veterinarias, Universidad de Chile, Santiago 8820808, Chile; prhuenchullan@gmail.com; 2 Laboratory of Veterinary Vaccines, Department of Animal Biology, Faculty of Veterinary and Animal Science, Universidad de Chile, Santiago 8820808, Chile; svidalvilches@gmail.com; 3 Departamento de Ciencias Animales, Facultad de Agronomía e Ingeniería Forestal de la Universidad Católica de Chile, Avenida Vicuña Mackenna 4860, Santiago 7820436, Chile; larrain@uc.cl

**Keywords:** immunocastration, recombinant vaccine, GnRH antibodies, meat quality, livestock

## Abstract

**Simple Summary:**

Castration of males is a common procedure in cattle production. Surgical procedures are most commonly used, but there is an increasing interest in non-invasive alternatives to avoid risk of infection, bleeding, pain, stress and to improve animal welfare. Immunization against gonadotropin-releasing hormone is currently being used in livestock, but there is only one commercially available vaccine for cattle and results regarding the number of doses needed to maintain castration are variable. The efficacy, safety, and production parameters of a new antigen for immunocastration in bulls was assessed. Results showed that two doses of the vaccine to 40 10-month-old bulls achieved testosterone suppression below productive performance. Live weight at slaughter and carcass yield was greater in immunized animals than in surgically castrated cattle. Castration effects of the vaccine were maintained until the end of the trial at 24 weeks.

**Abstract:**

Castration by surgical techniques is common in livestock; however, post-surgery complications and concerns for animal wellbeing have created a need for new non-invasive alternatives. The objective of this study was to evaluate immunocastration in bulls using antigen GnRX G/Q; a recombinant peptide proved to be effective in laboratory and companion animals. A nine-month trial with 80 9-month-old Normand x Hereford bulls, kept in a pastured system, was conducted. The herd was divided in half with 40 bulls surgically castrated (SC) and 40 castrated by immunization against GnRH (IC). The antigen was injected on days 0 and 40 of the experiment. After the second dose, the IC group had elevated GnRH antibodies and decreased testosterone levels (below 5 ng/mL) that were maintained for 23 weeks. At slaughter on day 190, the immunocastrated group obtained a higher weight, hot carcass, and dressing percentage than the SC group. There was no difference in pH, color of meat, fat coverage, cooking loss, or tenderness between groups. The bulls showed no inflammatory reaction at the injection site or adverse side effects from the vaccine. Our results demonstrate that immunocastration with GnRX G/Q is an efficient and safe alternative to surgical castration in livestock. Additional work evaluating antigen effects over a longer period is needed to validate commercial viability.

## 1. Introduction

In beef cattle production, it is important to reach optimal efficiency in feed conversion at the lowest possible cost. External disturbances such as sexual and aggressive sexual behavior could affect feeding conversion, resulting in production losses [1]. Because of this, castration is commonly used in livestock production to reduce the unwanted effects of handling uncastrated males [2] and to improve meat quality [3,4,5]. To date, surgical castration is the most commonly used technique by farmers worldwide. However, disadvantages of surgical castration are wound infections, stress in animals, hazards for medical staff [6], and the impact on animal well-being. An alternative non-invasive technique is immunocastration through vaccination against the endogenous gonadotrophin-releasing hormone (GnRH). The vaccine blocks native GnRH from binding to GnRH receptors in the anterior pituitary gland, suppressing gonadotropin secretion and inhibiting gametogenesis and steroid production as well as reproductive behavior [7]. This process achieves similar physiological and productive effects as surgical castration, without the adverse consequences to the animal’s well-being [8,9,10].

Several studies have evaluated the efficiency of immunocastration in cattle, pigs, and other species comparing different productive and reproductive aspects [5,8,11,12]. Despite some effectiveness, most vaccines only induce short-term results [3,13], and in some cases, cause adverse side effects from components of the formulation [14]. Some vaccines are based mainly on a carrier protein of hemocyanin, bovine albumin, ovalbumin, or tetanus toxoid fragment diluted in an aqueous adjuvant. To be effective, two or three doses of vaccine is given over four weeks [15]. There is only one commercially available vaccine for bovines, and it requires more than three doses to reach desired results [8,13,16], which may prevent this method from being a large-scale substitute to surgical castration.

GnRX G/Q is a recombinant protein vaccine with a tandem repeat primary structure developed by the Laboratory of Veterinary Vaccines of the Universidad de Chile. The vaccine does not need a carrier protein but only an adequate adjuvant to enhance its immunogenicity [17]. Studies have demonstrated the efficiency and safety of this vaccine both in laboratory and companion animals [17,18,19,20]. As for livestock animals, ongoing work has shown that the vaccine is safe and effective for achieving castration in pigs, without detrimental effects on the quality of meat [21].

The aim of this work was to compare surgical castration in cattle to the efficacy of immunocastration with the GnRX G/Q recombinant vaccine in regard to length of time of GnRH suppression, safety, production yield, and meat quality.

## 2. Materials and Methods

### 2.1. Vaccine Formula

The GnRXG/Q antigen is a recombinant protein of 23 kDa. It is a fusion protein composed of the primary amino acid sequence of the GnRH-I hormone fused to a spacer sequence that gives it immunogenic capacity [20]. The recombinant antigen was expressed in inclusion bodies of apathogenic *E. coli* bacteria strain BL21 (DE3) and purified from the bodies of inclusion. Since the GnRH hormone has low immunogenicity, the use of adjuvants is required. Low molecular weight chitosan (Sigma Aldrich, St. Louis, MO, USA) was used as an adjuvant in this trial [18]. The final formulation included 500 µg of antigen and 500 mg of chitosan in 2 mL, final volume.

### 2.2. Animals and Handling

The clinical study was conducted in a commercial system based on a meadow environment at Huifquenco Farm in the region of Araucanía, municipality of Villarrica, south of Chile. Eighty Normand × Hereford intact males arrived at the farm at 9 months old and with a live weight of 196 ± 25.2 kg (mean ± SD). Animals were fed improved natural meadows (9000–11,000 kg/dm); mainly ryegrass and white clover pasture. At 10 months of age, animals were randomly assigned to two treatment groups of 40 individuals each: surgically castrated (SC) and immunocastrated (IC) with anti GnRX G/Q vaccine. Both groups were fed for 240 days (8 months), from August 2015 to March 2016, in the same four-hectare paddock area, rotating every 3 days according to the growth of the meadow. All animals were sent to slaughter at 18 months of age. Recording and analysis of the variables was blind to avoid influence from the observer. The study was conducted under the permission of the bioethics committee of the Universidad de Chile (Certificate N°12−2014).

### 2.3. Castration

#### 2.3.1. Surgical Castration

All animals in the control group were castrated at 11 months of age with an average live weight of 244.33 ± 32.70 kg. Surgical castration was performed as follows: Xylazine hydrochloride (Xila10^®^, Santiago, Chile) was applied at a rate of 0.3 mg/kg intramuscularly; after 5 min, the animal was restrained in a chute, and a knife disinfected with trypaflavine was used for scrotal incision. The testicles were pulled and cut at the spermatic cord using an emasculator. No analgesia was used, in accordance with the common practice in Chile. Finally, a larvicide, repellent, and antiseptic spray (Larvicida Spray^®^, Santiago, Chile) was sprayed from 10 to 20 cm to cover the entire affected area, including the surrounding healthy area.

#### 2.3.2. Immunocastration

The IC group received the first dose of GnRXG/Q two days before surgical castration of the SC group. The vaccine was administered in two doses: a first vaccination (set as day 0 of the study) at 11 months of age (average weight of 243.19 ± 35.06 kg) followed by a booster at day 40 (average weight of 289.08 ± 39.06 kg). A sterilized 18-g needle inserted at 45° subcutaneously toward the cranial on the right side of the neck was used in accordance with recommendations from the laboratory. Prior to injection, a dry, clean section was disinfected with 70% (*v*/*v*) ethyl alcohol, sliding cotton swabs cranial to caudal three to four times, one pass per swab.

### 2.4. Safety of Vaccine

After vaccination, animals were inspected at 6, 12, 24, and 48 h to assess the appearance of symptoms at the injection site using a scoring system for beef cattle created for this purpose, in which the injection site, general condition, and behavior are evaluated [22] (Table 1). According to the protocol, the general condition of each animal was evaluated by farm workers every 12 h until the end of the study.

### 2.5. Blood Samples

A subsample of 10 bulls in each group (*n* = 10) was randomly selected for blood sampling. To obtain serum, 10 mL of blood were sampled by jugular venipuncture from all animals on days −30 (30 days before vaccination), 40, 70, 120, and 170 (days after first vaccination; booster was on day 40). On the same day, serum was separated from clotted blood by centrifugation at 3000 rpm for 10 min, aliquoted in 2 mL Eppendorf tubes and frozen at −20 °C until analysis.

### 2.6. Specific Immunoglobulin Assay

According to method described by Siel et al. [17,18,19], an indirect ELISA assay was performed in 96-well plates (MaxiSorp, Nunc, Darmstadt, Germany) to detect the concentration of specific antibodies (IgG) against the recombinant protein of the inoculated animals. Two μg of GnRX G/Q and fifty μL of adhesion buffer (150 mM Na2CO3, 350 mM NaHCO3, pH 9.6) were added to each well and incubated for 2 h. Plates were then washed 5× with wash buffer (Tween-20 0.05% *v/v* in PBS) and blocked with 200 μL of blocking buffer (Bovine serum albumin 1% *w/v* in PBS) overnight at 4 °C. The plates were then washed 5× with wash buffer and incubated with 150 μL of each serum diluted 1:250 in dilution buffer (BSA 0.1% w/v, Tween-20 0.05% v/v) for one hour at 37 °C. Finally, the plates were washed 5× with wash buffer and incubated with 150 μL of Rabbit anti-Cow IgG peroxidase-conjugated antibodies (Jackson Immunoresearch Laboratories) diluted 1:5000 in dilution buffer for 1 h at 37 °C, then rewashed and developed with 150 μL of 1-Step™, Slow TMB-ELISA (Pierce, Chemical Company, Dallas, TX, USA) for 15 min at room temperature. The reaction was terminated with 150 μL of 1.5M H2SO4 and the absorbance was read at 450 nm.

### 2.7. Testosterone Assay

A Competitive Enzyme Immunoassay (DRG International, Inc., Springfield, MO, USA) was performed on the serum of both SC and IC groups in accordance with the manufacturer’s recommendations. Briefly, 96-well plates precoated with pre-blocked anti-rabbit monoclonal mouse IgG were incubated with experimental animal serum (1:50) in conjunction with Testosterone-bound Acetylcholinesterase and rabbit antiserum testosterone in EIA buffer (Cayman Chemical Company, Ann Arbor, MI, USA) for two hours at room temperature. Results were read at 405 nm after a one-hour incubation with Ellman’s Reagent (Cayman Chemical Company), according to the manufacturer’s recommendations (catalog EIA-155996).

### 2.8. Production Indicators and Meat Quality

#### 2.8.1. Preslaughter Measurements

Production parameters of immunocastrated versus surgically castrated males (*n* = 40) were evaluated throughout the trial. Live weights (LW) were measured on days −30, 0, 40, 70, 120, 170, and 190 (counting day 0 as the day of vaccination and day 40 as the booster). Average daily gain (ADG) was calculated with weight measurements between the onset of the study and primary (first) vaccination, between the primary vaccination and the booster (second), and from the booster to day 190 (final). On day 190, all animals were transported to the slaughterhouse (“Frigorífico Temuco”). Animals arrived at the end of the day (8:00 PM) and were, therefore, kept waiting in pens until early hours the next day. All animals were slaughtered in a single batch between 06:00 and 07:30 am.

#### 2.8.2. Carcass Evaluation

Slaughter was carried out under standard Chilean protocol and was inspected by the Agricultural and Livestock Service (SAG) of the country. Once the carcass of the animal was obtained, it was taken to a cold chamber (−4 °C) until analyses 48 h later. After slaughter, carcasses were measured for weight of the hot carcass (HCW), dressing percentage, and fat cover by the Certification Department of the slaughterhouse. Briefly, HCW = carcass weight after evisceration; dressing percentage = percentage of the hot carcass weight with respect to the live weight; fat coverage scored on a scale of 0 to 3, where 0 = no fat cover, 1, 2 = medium cover, 3 = oiled. Additionally, carcasses were classified by category following Chilean regulations (Law No. 19.162 of 1992). This is the final classification given to a carcass according to age, dental chronometry, and fat coverage. The classification follows the Spanish acronym VACUNO (which means “bovine”): V = young steer, heifer, young bull (2 maximum teeth) and calf (maximum milk teeth), fat coverage 1–2; A = steers and young cow (4 teeth maximum and 1–2–3 fat coverage); C = steers and young cow (6 teeth maximum and 1–2–3 fat coverage); U = adult cow, ox or bull (8 teeth or full mouth and fat cover without requirement); N = old cow, ox, bull (teeth = from leveling of the second medians and fat coverage without requirement); O = calf and veal (teeth = without leveling the milk centers or tweezers and fat coverage without requirement).

The pH was measured 24 h post-mortem (pH24) in the muscle *Longissimus thoracis* (LT) between the 9th and 10th ribs, using a pH meter and a thermometer (Hanna^®^, Woonsocket, RI, USA). Carcasses with pH24 values above 5.8 were considered dark cutting or DFD (dark, firm, and dry), which is correlated with a dislike of the meat by consumers [23].

#### 2.8.3. Measurement of Meat Quality

Analyses were carried out in the Meat Quality Laboratory of the Pontificia Universidad Católica de Chile. A subset of animals (*n* = 7 for each group) was randomly selected for meat quality measurements. The number of animals in each group was determined based on results from Larraín et al. [24] for Warner-Bratzler shear measurements (coefficient of variation around 7%), 90% power at *p* < 0.05, and an expected difference to be detected between treatments of 15% (six replicates needed according to Bernston [25]). Forty-eight hours after slaughter, a 2.5-cm steak was cut from the LT of each carcass, vacuum-packaged and kept frozen at −18 °C until analysis. In the laboratory, samples were thawed at 4 °C, removed from the vacuum packaging, and allowed to bloom for 30 min before measuring color with a chroma meter (Minolta CR-400, Osaka, Japan), previously calibrated with a white calibration plate (L* = 97.06, a* = 0.14, b* = 1.93). Data were collected in CIE L*a*b* color space. Lightness (L*), redness (a*), yellowness (b*), chroma (or color saturation), and hue angle (arctangent (b*/a*) × 360°/(2 × 3.14)) were evaluated. The color coordinates for each sample were calculated as the average of three measurements.

To measure weight loss after cooking (CL), samples were cooked in an open-hearth electric grill. Steaks were flipped when internal temperature, monitored with a portable meat thermometer, reached 40 °C at the geometrical center [26]. Cooking was stopped when the temperature reached 71 °C and the samples were cooled at room temperature. Cooking loss of weight was calculated by differences in weight of samples before and after cooking.

Shear force was measured by removing six cylindrical cores per sample of 1 cm in diameter with a press, parallel to the muscle fiber orientation. Each piece was refrigerated and sheared perpendicular to the muscle fiber orientation when it reached 4 °C. Measurements were made with a tabletop Warner-Bratzler (WB) shear machine (G-R Electrical Manufacturing Co., Manhattan, KS, USA) at a speed of 3.3 × 10^−3^ m/s. The tenderness was calculated as the average measure of six cores [26].

### 2.9. Statistics

Statistical analysis was completed using GraphPad PRISM 7.0. All variables were treated the same. The Shapiro–Wilk test was used to verify if variables had a normal distribution. If normally distributed, the F test in the ANOVA was used. When the null hypothesis of equality of mean effects was rejected (*p* < 0.05), Tukey’s or Bonferroni’s multiple comparison procedures were used. For variables that did not behave in a normal way (cooking and color), the Kruskal–Wallis test was applied.

## 3. Results

### 3.1. Evaluation of Vaccine Safety

None of the 40 bulls vaccinated in the trial presented any degree of injury or general compromise (weight loss, abnormal behavior, and/or difference in the appearance of their coat). According to the scoring system used (Table 1), no adverse reactions were recorded at the injection site at 6, 12, 24, and 48 h post-injection.

### 3.2. Immune Response

**Figure 1:**
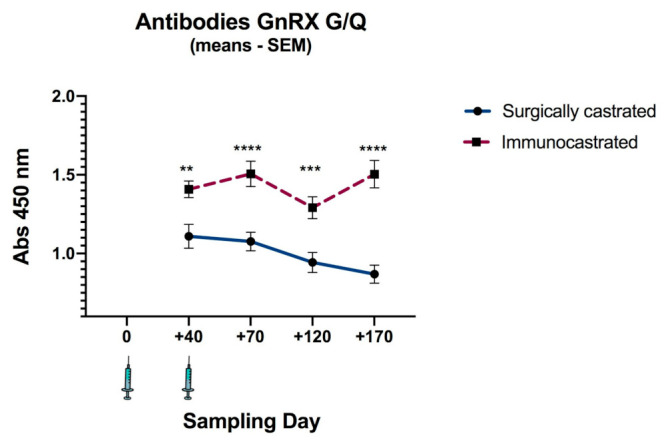
Production of anti GnRXG/Q IgG by immunocastrated (IC) Norman × Hereford bulls compared to surgically castrated (SC). The IC group was vaccinated on day 0 and 40 with GnRXG/Q antigen. Data represented as mean ± SEM. Asterisks indicate intergroup significant difference (*p*-values: * <0.05; ** <0.01; *** <0.001; **** <0.0001).

After the first vaccination (Day 70), the IC group had increased anti GnRXG/Q IgG production significantly different than the SC group (*p* < 0.0001). The difference remained until the end of the study (Figure 1). An antibody drop was observed on day 120. Afterward, antibody concentrations rebounded and the statistical difference between IC and SC increased, showing that the vaccine is able to maintain immunocastration for at least 170 days (24 weeks).

### 3.3. Testosterone Suppression

**Figure 2:**
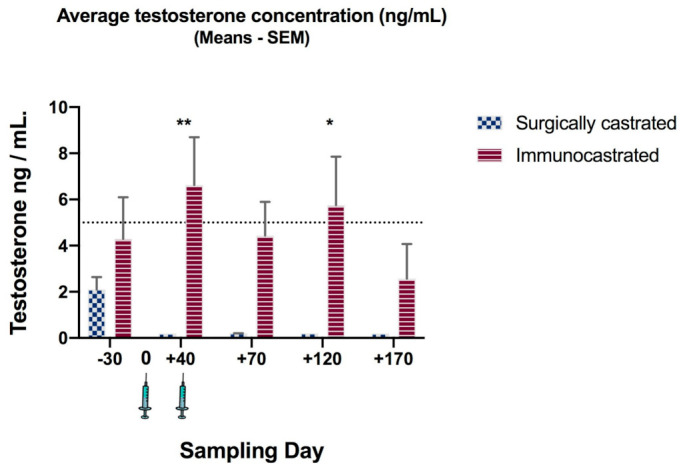
Average testosterone concentration (ng/mL) for Norman × Hereford bulls surgically castrated (SC) and immunocastrated (IC). Superscripts indicate significant difference (*p*-value: * < 0.05; **< 0.01).

**Figure 3:**
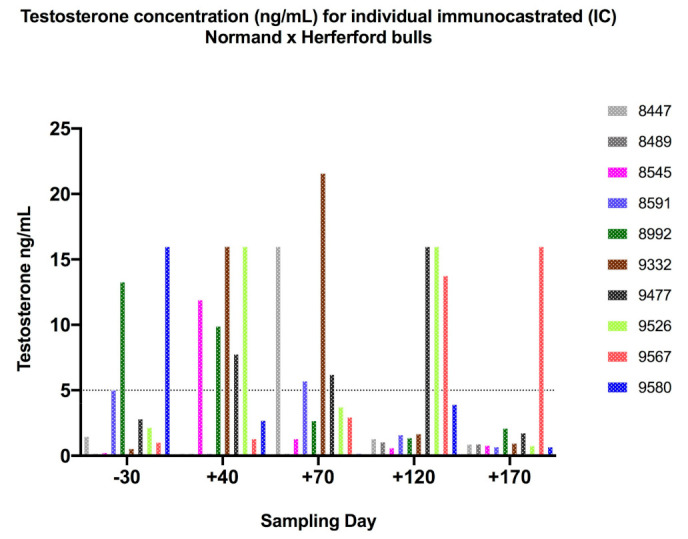
Individual testosterone concentration (ng/mL) for 10 immunocastrated (IC) Norman × Hereford bulls. Each color represents one bull sampled at various time points pre- and post-vaccination from Day −30 to Day 170.

No difference in mean testosterone concentrations between control and vaccination groups was observed at the beginning of the trial. Values were within the expected range in pubertal cattle (Figure 2). The IC group had significantly higher testosterone concentrations than the SC group until Day 40 (*p* < 0.01). Following the booster, IC testosterone concentrations fell below 5 ng/mL at days 70 and 170, indicating that immunocastration with GnRXG/Q reduced testosterone to a level similar to surgical castration. When analyzing individual animals, we observed that the vaccination was effective in 50% of the individuals after the first dose at day + 40 (concentrations ≤ 5 ng/mL), ultimately reaching a higher, 90%, effectivity by day + 170 (Figure 3).

### 3.4. Production Parameters and Meat Quality

No differences in ADG at the first or second vaccination were observed between SC and IC (Table 2). However, there was a significant difference in final ADG, which led to a difference in LW on days 170 and 190 (*p* < 0.01). At the slaughterhouse, weight of the hot carcass, dressing percentage, pH, fat cover, and category were determined (Table 2). The IC group had a greater weight (*p* < 0.01) and carcass yield (*p* < 0.05) than the SC group. No significant differences were observed in the fat coverage, color, and pH. Both groups were classified as “V” meat under Chilean quality classification (i.e., cattle less than 2 years old and fat coverage between 1 and 2). There were no differences between groups for quality of meat (Table 3).

## 4. Discussion

### 4.1. Effectivity of the Vaccine

This study reports the first use of recombinant vaccine GnRXG/Q in Bos Taurus bulls. After two doses of vaccine, through day 170, bulls showed significantly higher levels of GnRX G/Q antibodies than the SC group (*p* < 0.001). Higher antibodies levels were associated with low concentrations of testosterone, which fell below a threshold of 5 ng/mL after the booster vaccine. The immune response and testosterone suppression was maintained until the end of the trial at 24 weeks. The antibody response pattern after the first and second dose is similar to those previously obtained in bull calves and dairy cattle using different vaccine formulations: a slight increase in antibodies after the first vaccination and a robust response after the second dose [27,28,29,30,31]. The slow immune response and slight effect on testosterone suppression after the first dose may allow producers to take advantage of the anabolic effect of testosterone in bulls, as noted by Amatayakul-Chantler et al. [29]. Similar results were found in dogs and mice (female and male) [17,19]. Significant differences in the level of specific antibodies against GnRX G/Q after the first immunization could lead to later immunization strategies; potentially resulting in increased growth in cattle due to the anabolic effects of testosterone.

Amatayakul-Chantler et al. [29] measured low antibody concentrations at day 60 post-first vaccination and claimed that the immune response decreased over time, making a third or fourth dose necessary to obtain an effective response [32]. However, they used Bopriva^®^, a vaccine based on a carrier protein, known to induce rapid immune tolerance in the organism [18,33]. In this study, there were no decreases in IgG, and after the booster, antibody concentrations stayed around 1.5 OD 450 nm. The immune response depends on several factors, such as route of inoculation, number of doses, and active components and adjuvants of vaccine [17,34]. The difference between studies could be attributed to our recombinant antigen; a possible molecular pattern associated with pathogens (PAMP) may have improved the immunogenicity of the GnRX G/Q vaccine [5]. Furthermore, previous research in our laboratory has shown that chitosan adjuvant can maintain high levels of GnRX G/Q Ab up to 7 months after first vaccination [17].

Both groups were positive for anti GnRX G/Q IgG. Improved standardization of the in-house technique used for antibody analyses is necessary for further assays. Similar results in previous studies with the GnRX G/Q vaccine in mice [19] and pigs [21] in the baseline measurement and those obtained by Amatayakul-Chantler et al. [5] in bovine have been reported. Nevertheless, significant differences of anti GnRX G/Q between IC and SC demonstrate the persistence of antibodies through time, which is correlated to levels of testosterone in this assay, similar to results obtained by Siel et al. [19]. The temporary drop of antibodies at day 120 may be the result of individual variability as suggested by Theubet et al. [35]. An increase of testosterone at day 120 correlates with the antibody drop, supporting the association between antigen and hormone suppression. After day 120, there was a rebound in antibody concentration and the statistical difference between the IC and SC group increased. The vaccine is able to keep the animals immunocastrated for at least 161 days (23 weeks).

At the beginning of the study, there was no difference in the mean testosterone concentrations between both groups. Levels were within the expected range in pubertal cattle. In accordance with previous studies, low testosterone levels in the IC group (below threshold of 5 ng/mL for entire bulls) were associated with high levels of GnRH antibodies [5,27,30], showing that this vaccine is effective for immunocastration. Our results indicate that only two doses reduced testosterone to a similar level as surgical castration. Other vaccines with different adjuvants applied to *Bos taurus* and *Bos indicus*, in feedlot and pasture, respectively, needed three–four doses to maintain testosterone suppression for 15 weeks [8,13,16,36]. When analyzed individually, this vaccine was able to decrease testosterone production in 50% of the individuals after the first dose at day + 40 (concentrations ≤ 5.00 ng/mL) and in almost 90% of individuals on day +170 (Figure 3). It is known that serum testosterone levels in bovines begin to increase linearly between 4.3 and 7.3 ng/mL from 7 to 13 months of age [37] and is highly variable between individuals [38]. This could explain the variability of testosterone concentrations in this study, since the animals started the experiment at 11 months of age with concentrations between 0.19–16.01 ng/mL (coefficient of variation = 32.67% in the IC). Additionally, the immune response to vaccination depends on a variety of factors and genetics may play a role in diminished or nonresponsive individuals [20,39]. Therefore, individual variability should be considered when evaluating the response to immunization against GnRH. In this study, the average level of testosterone was below what is considered normal for males; therefore, it is assumed that in the vaccinated individuals, the use of the vaccine was an effective method of castration [29]. Moreover, it could be hypothesized that inhibition of testosterone production would also suppress unwanted behaviors, as reported previously [3,40]. Further studies on behavior are needed to test this hypothesis.

### 4.2. Performance and Quality of Meat

The growth performance of animals castrated with the GnRXG/Q vaccine was at least equal to those surgically castrated. For several measurements, IC performed better the SC group. The final ADG was higher in IC than SC animals, explaining the higher final LW in the IC group. While three ADG measurements were taken at different time points, only the final ADG measurement coincided with the period of fastest growth. These results are similar to previous studies in a grazing Nellore feedlot, where IC and SC were compared [5]. Results for ADG with and without vaccination are highly variable. Previous pasture studies on Bos indicus [13,16] and Bos Taurus in a feedlot [8] showed no differences in live weight between immunocastrated and surgically castrated animals. The authors argued that the variability of the results between the studies could be explained by the stress to which the castrated animals are subjected, decreasing their weight gain and final growth without being able to recover, as shown by Bretschneider [9]. Differences between studies could be influenced by the age at which animals are castrated. Older animals are more susceptible to significant weight loss and stress, as suggested by Noya, Ripoll, Casasús, and Sanz [41]. In agreement with Amatayakul-Chantler et al. [5], the greater final LW of immunocastrated bulls in this study seems to corroborate the advantages of immunocastration over surgery.

The immunocastrated bulls obtained a higher HCW than the SC group. This effect is important as HCW it is the critical feature for which producers are paid. Although previous results have shown an advantage in growth performance of surgically castrated over immunocastrated males [8,42], our results agree with Amatayakul-Chantler et al. [5]. The crossbred cattle used in this study might have influenced the results. According to Miguel et al. [43], immunocastration generates greater productive benefits in crossbred cattle. In this study, Normand x Hereford bulls immunocastrated with the GnRX G/Q vaccine have a significantly better HCW performance and dressing percentage than surgically castrated animals. While breed could influence the results in this work, it is important to highlight that slight levels of testosterone in the bulls of the IC group might influence growth.

The average pH for both groups was 6. This parameter is evaluated in all slaughterhouses of Chile to determine whether the quality of the meat is suitable for exportation. Dark cuts are not only unpleasant for consumers, but also implies a reduced shelf life. In Chile, and in agreement with USDA grading standards, carcasses with pH values of 5.8 or greater are considered DFD [44]. Final pH depends on the glycogen reserves contained in the skeletal muscle, which highly depends on the stress of the animal prior to slaughter. It is known that the longer the transport and waiting times for the animals to be slaughtered, the higher the probability of obtaining dark cuts of meat, due to the energy expenditure involved [45]. In our study, the long period that animals remained in the slaughterhouse pens could have influenced results [44,45]. Our work did not evaluate the difference in pH between immunocastrated and whole bulls, but others have reported that vaccination does not affect pH [29,32,46].

There were no differences between the SC and the IC in fat coverage (Table 2) and all carcasses were classified with the highest score (“V” category), according to the Chilean scale, indicating the same fat percentage and lack of lesions in both groups. This differed with work carried out by Amatayakul-Chandler et al. [29], where immunocastration improved fat coverage according to USDA carcass grading. Diet may explain the differences between the studies as in this study, cattle were grazing, and in Amatayakul-Chandler et al., cattle received a feed-lot diet of corn and grains.

The results in this study show that this vaccine does not have a detrimental effect on meat quality. There were no significant differences between tenderness, cooking loss, and color, similarly to that seen by others [5,16,43]. Bright red color in meat is a desirable attribute for commercialization and is the most relevant parameter for fresh meat [47]. In this study, there were no differences in instrumental color of meat between IC and SC.

None of the 80 cattle used in the trial presented any degree of injury or general compromise (weight loss, abnormal behavior, and/or difference in the appearance of their coat). These results agree with those provided by other studies regarding the safety of the GnRX G/Q vaccine in other species [19,20,21].

## 5. Conclusions

Administration of two doses of recombinant vaccine GnRX G/Q achieves a reduction of testosterone below 5 ng/mL for at least 23 weeks following the first dose of antigen. Importantly, the effects on production parameters such as average daily gain, fat coverage, and meat quality are the same as animals surgically castrated. Moreover, bulls that received the vaccine were heavier than surgically castrated animals at the end of the trial and had better HCW and dressing percentage, probably because of their low levels of testosterone throughout the trial. The stress of castration may have influenced the lower final weight of the SC group. Although behavior was not studied in this work, reduction of hormone levels and growth performance suggest that the vaccine accomplished the effects of castration in suppressing undesirable behavior that has detrimental effects on production.

Our results provide evidence that antigen GnRX G/Q is a safe and efficient alternative to surgical castration in cattle. Longer trials are needed to determine the maximal effects of the vaccine for commercial proposes, i.e., suitability for use in beef cattle production with longer feeding times.

## Figures and Tables

**Table 1:** Security score for assessment of adverse reactions after vaccination.

Category	Variables	Score
Injection Site (0–3)	No reaction	0
	Swelling, but not abscessed	1
	Non-sore abscess	2
	Sore abscess	3
Weight loss (0–4)	Normal (without weight loss)	0
	Slight weight loss (less than 10%)	1
	Abnormal appearance/amount of feces	2
	Weight loss (more than 20%)	3
	The animal does not consume water or eat	4
Appearance (0–3)	Normal	0
	Bad hair appearance	1
	Eye or nasal discharge	2
	Abnormal Posture	3
Normal Behaviour (0–4)	Normal	0
	Inactive stand or lying	1
	Abnormal vocalizations	2
	Inactive stand and bent back	3
	Lying down with abdominal breathing	4

Note: when an animal scores 3 in more than one parameter, all “3s” move to “4.” The suggested corrective measures based on the score obtained for each animal are the following: 0–2, Normal; 3–7, Carefully monitor (analgesics); 8–10, Severe suffering—separate and add analgesics; 11–14, Euthanasia.

**Table 2:** Production parameters of surgically castrated (SC) and immunocastrated (IC) cattle.

Parameter	SC Mean ± SEM	IC Mean ± SEM	*p*-Value
LW initial (kg)	194.5 ± 4.10 (*n* = 40)	197.5 ± 4.0 (*n* = 40)	ns
LW d 170 (kg)	353 ^a^ ± 4.3 (*n* = 40)	377 ^b^ ± 5.45 (*n* = 40)	<0.01
LW d 190 (kg)	428 ^a^ ± 5.0 (*n* = 40)	450 ^b^ ± 6.5 (*n* = 40)	<0.01
ADG 1st (kg/d)	1.46 ± 0.10 (*n* = 40)	1.44 ± 0.07 (*n* = 40)	ns
ADG 2nd (kg/d)	0.99 ± 0.05 (*n* = 40)	1.09 ± 0.06 (*n* = 40)	ns
ADG final (kg/d)	1.75 ± 0.02 (*n* = 40)	1.84 ± 0.03 (*n* = 40)	<0.01
HCW (kg)	217 ^a^ ± 3.2 (*n* = 20)	237 ^b^ ± 3.5 (*n* = 20)	<0.05
Dressing percentage (%)	51 ^a^ ± 0.4 (*n* = 20)	53 ^b^ ± 0.4 (*n* = 20)	<0.05
pH 24 h	6.0 ± 0.05 (*n* = 20)	6.0 ± 0.04 (*n* = 20)	ns
Fat coverage	1 (*n* = 20)	1 (*n* = 20)	ns
Category	V (*n* = 20)	V (*n* = 20)	ns

Live weight (LW) at the beginning of the trial and on days 170 and 190; ADG average daily weight gain between the onset of the study and primary vaccination (first), between the primary vaccination and the booster (second), and from the booster to day 190 (final); Hot carcass weight (HCW, carcass weight without viscera, leather, head, legs, and tail); Dressing percentage (HCW/LW expressed as percentage); Fat coverage (score scale from 1 to 3) and pH at 24 h post-mortem. Differences in the number of animals for HCW, dressing percentage, pH, and fat coverage are due to missing data from the slaughterhouse. Category according to Chilean classification. ns = not significant (*p*-value > 0.05).

**Table 3:** Characteristics of meat quality of immunocastrated (IC) and surgically castrated (SC) cattle.

Variable	SC Mean ± SE	IC Mean ± SE	*p* < 0.05
Cooking loss (%)	14.16 ± 11.0	17.22 ± 18.0	ns
Shear Force (Kg)	2.2 ± 0.36	2.0 ± 0.18	ns
L *	35.43 ± 2.50	38.02 ± 1.40	ns
a *	19.36 ± 2.80	20.37 ± 1.70	ns
b *	10.40 ± 2.14	11.60 ± 1.43	ns
H *	0.37 ± 0.05	0.32 ± 0.01	ns
C *	297.43 ± 49.10	319.33 ± 26.90	ns

L = luminosity: a * = redness; b * = yellowness; H * = Hue angle; C = chroma. ns = not significant (*p*-value > 0.05).

## Data Availability

The data used and analyzed during paper preparation are available from the corresponding author on reasonable request.
